# Appreciation of a Child's Journey: Implementation of a Cardiac Action Research Project

**DOI:** 10.1155/2012/145030

**Published:** 2012-04-29

**Authors:** Kate Alexa Dengler, Valerie Wilson, Sarah Redshaw, Gabrielle Scarfe

**Affiliations:** ^1^Edgar Stephens Ward, The Children's Hospital at Westmead, Sydney, NSW 2145, Australia; ^2^Nursing Research and Practice Development Unit, Kids Research Institute, The Children's Hospital at Westmead, Locked Bag 4001, Westmead, NSW 2145, Australia; ^3^Faculty of Nursing, Midwifery and Health, University of Technology, Sydney, NSW 2007, Australia

## Abstract

The aim of this paper is to provide an overview of the phases of the action research process involved in developing, implementing, and evaluating the Heart Beads program. The aim of the project is to enrich the hospital experience of children with cardiac conditions. Heart Beads involves children receiving unique beads specific to each cardiac treatment, procedure or event in recognition of their experiences, and endurance. An action research approach, involving a partnership between clinicians and researchers and emphasising the involvement of patients and their families, was used to guide the Heart Beads program. The project followed the five phases of action research: identification, investigation, program development, implementation, and evaluation. Heart Beads began as a small project which continues to grow in popularity and significance with children at a tertiary paediatric hospital in Sydney, Australia. The program is now being implemented nationwide with the vision that all Australian children hospitalised with cardiac conditions can benefit from Heart Beads.

## 1. Introduction

In July 2008, The Children's Hospital at Westmead, Sydney launched the Heart Beads program for children with cardiac conditions. The program involves children receiving unique beads specific to each cardiac treatment, procedure, or event in recognition of their experiences and endurance. The program aim was to document the children's experiences in a unique and child-friendly form, adding a positive dimension to their treatment process and ultimately reducing the strain involved in their hospital admission. The project team (nursing staff and external researchers) hoped that children would feel a sense of achievement for their courage and be able to trace their continuing journey as their collection became increasingly unique and also that the beads could act as a medium for the children and families to develop more positive relationships with staff and other children and families in the hospital.

Heart Beads was developed using an action research approach to help address the needs of children, families, and staff identified both within the hospital and the existing literature. The stresses parents experience when a child is diagnosed with a serious illness is well documented [1–5]. The literature also details children's need for information about their hospitalisation [6], and the importance of children being spoken to and informed about their illness and treatment [7]. The provision of information has been identified in a number of studies as the primary need in coming to terms with a chronic condition and in involving children in their own care [6, 8, 9]. Communication and negotiation over a child's care between nurses and parents has been found to be important in family-centred care though it is often found to be inconsistent and is in need of further exploration [10]. Communication between health professionals and parents can impact on psychological distress at the time of diagnosis and treatment, and communication between parent and child in the future [11]. Previous research on the cardiac ward had identified the impact of such stressors [12] and was one of the motivators for the development of a program that would acknowledge and honour the courage of children with cardiac conditions.

Action research has been widely used in nursing and other disciplines to help guide the development of new programs, the introduction of these programs into clinical settings, and the subsequent evaluation of the program [13–20]. There are various types of action research. The action research approach involves a spiral of self-reflective cycles with each step evaluated as the process proceeds [21]. This approach brings together action, reflection, theory, and practice in a systematic way in order to create practical knowledge that is of value to those involved in the focus of the research [22]. Action research allows knowledge development which can be applied back to the practice area from which it was created [19]. An advantage of an action research approach is the collaborative relationship between the researchers, staff, patients, and families in the clinical setting that can enable sustainable changes to patient care and the work environment [23].

The aim of this paper is to provide an overview of the phases of the action research process involved in developing, implementing, and evaluating the Heart Beads program created with the aim of enriching the hospital experience of children with cardiac conditions.

## 2. Design and Methods

An action research approach was used to guide the development, implementation, and evaluation of the Heart Beads program. This approach was deemed the most appropriate due to the facilitative nature of action research and the fact that staff were familiar with action research processes. Such an approach is important as the program was aimed not only at improving the experience for children, young people, and their families but also included supporting clinical staff to undertake and learn about research, to make changes to practice, to evaluate the outcomes of such changes, and to report the findings to a range of audiences [24]. Meetings were regularly held on the ward with both the nursing staff and researchers throughout the planning, implementation, and evaluation of the program. The action research cycle involves phases whereby evaluation is continuous, and changes are integrated in different phases of the process [25, 26]. Numerous program changes arose as a result of continual stakeholder feedback and evaluation. 

**Figure 1:**
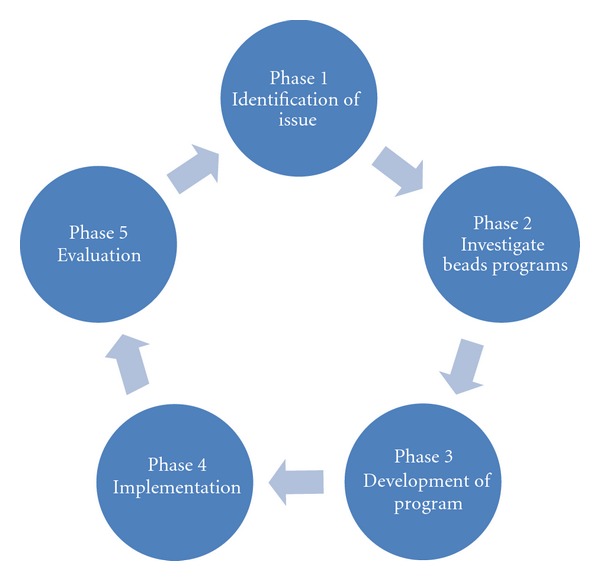
Action research phases for the Heart Beads project.

 This project followed the five phases of action research (Figure 1), as outlined by Wilson et al. [27].

- Phase 1 involved identification of the potential value of documenting children's and parent's experiences of hospitalisation for a cardiac condition.
- Phase 2 was the investigation of bead programs as a way of documenting the child's experiences as well as enabling staff to review and change their current practice.
- Phase 3 encompassed the program development which involved establishing a project team and planning the project including identification of elements and stages of the program.
- Phase 4 involved the clinical implementation of the program with the incorporation of various required changes in the process.
- Phase 5 was the overall evaluation of the program primarily through interviews with both staff and families.

The project phases discussed in this paper were conducted between 2007 and 2009. The Heart Beads program continues to be run at the hospital.

Ethics approval for the study was gained from the hospital Human Research Ethics Committee. Participation in the Heart Beads program is optional with the only inclusion criteria being that the child's primary reason for hospital admission is a cardiac condition. Written consent (for the child and their parents) is obtained from families who wish to participate in the program. Additional consent was sought for interviews and included consent from children and young people where they were able to be involved.

### 2.1. Phase 1: Identification

Practice Development projects conducted in the cardiac ward enabled staff to increase their awareness of the experiences of children, parents, and other staff and thus initiate improvement projects with the capacity for change at the ward level. During this phase ward staff saw a potential for recognition and documentation of children's and parents' experiences of hospitalisation. A bead program was identified as a project that could improve the experiences of cardiac patients and their families. Awareness of the potential value of bead programs in a health care setting was raised at a conference attended by the cardiac ward nursing unit manager (NUM) and cardiology clinical nurse consultant (CNC) at which “Bravery Beads,” a reward program for oncology children, was presented. The concept originated from the British Columbia's Children's Hospital, Vancouver where “Bravery Beads” was first developed as a tool to honour and document the journey of children with cancer [28]. Since the inception of this pioneering program, many similar programs have been implemented in various hospitals around the world for children with cancer. While the nursing staff were not aware of similar programs for children with cardiac conditions, it was thought that such a program might work to bring a sense of achievement and recognition to cardiac children.

### 2.2. Phase 2: Investigation

The cardiac ward NUM and cardiology CNC presented the bead concept to staff in the ward and staff in the nursing research and practice development unit. The program was subsequently discussed and investigated via literature search and communication with the developers of the Bravery Bead program. Whilst there are many anecdotal accounts of the Bravery Bead program and its benefits, it was found that there are to date no reported research studies on the implementation of such programs. There has, however, been research indicating the importance of finding ways to encourage children and prepare them for procedures they are about to undergo where possible [6]. A bead program seemed to offer a way to encourage children and positively document their cardiac procedures. This approach also enabled staff to review their current approach to procedures and to consider the implications of the bead program in changing their practice.

### 2.3. Phase 3: Program Development

**Figure 2:**
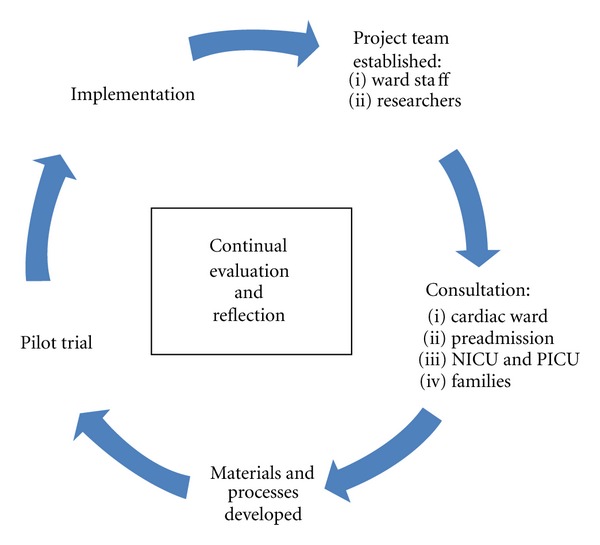
Research cycle for Heart Beads program development.

The development stage consisted of planning and designing the Heart Beads program. In this third phase a project team was established consisting of the project officer (the nurse educator on the cardiac ward), the cardiac ward NUM, cardiology CNC, other nurses on the cardiac ward, and researchers from the Nursing Research and Practice Development Unit at the hospital. This team met on a monthly basis to discuss project progress and issues arising and to evaluate the process and its evolution with staff. Program development, from initial funding approval to clinical implementation of the Heart Beads program, took approximately eight months. Program creation was multifaceted including developing staff education seminars, bead selection, brochure and logo development, structure and process development, formation of a risk management plan, development of patient and family consent forms and bead prescriptions, and stakeholder engagement and consultation. Not all aspects of program development were predictable, and some were only revealed as the program evolved. Further information on these aspects and overall program development are detailed in Dengler et al. [29]. Evaluation was continuous throughout the program development phase, and changes were integrated as the project progressed. Reflection and appraisal occurred at each stage of the program development and feedback from all relevant stakeholders taken into consideration. The process of Heart Beads program development is outlined in Figure 2.

Consultation took place with nursing and research staff, families, and representatives of the four hospital units where children with cardiac conditions reside: the cardiac ward, the preadmissions clinic and the Paediatric and Neonatal Intensive Care Units (PICU and NICU). The purpose of these consultations was to ensure that stakeholders including clinical staff and families were involved in the project from the beginning, and that the program was responsive to the needs of both staff and families. The procedures chosen to be represented through the beads to signify the most significant, traumatic, or memorable moments of a hospital stay were agreed upon. A pilot trial with one family was conducted with a sample set of beads. The five-year old patient was a long-term cardiac patient, thus relevant beads were backdated for previous events and procedures. The family's responses were sought by researchers during an interview. The family responded enthusiastically to the concept, the beads chosen and the processes and events they represented. The patient also featured in photos for Heart Beads brochures and other program material.

A process for dispensing the beads to children was developed to ensure safe distribution. The program was designed so that the workload of ward staff was kept to a minimum. After a process of trial and consultation with all stakeholders, a prescription idea was adapted from the “Bravery Bead” program. The onus was placed upon parents to carry a script and prompt staff when confirmation of an event was needed. Ultimately this meant that all that was required of the clinical staff was to initial and date a box on the child's distribution form once a procedure or event had occurred for which a bead could be dispensed. Due to procedure evaluation the script format changed to ensure that it was more effective and encompassed all relevant events and procedures.

This phase was important for establishing the project team (staff on the cardiac ward with external researchers) and their roles in the Heart Beads program which was based in the cardiac ward. Ongoing consultation between the project team and other units that were involved in the care of cardiac children and their families was also an important aspect. Processes and materials were continually refined as a result of constant evaluation throughout the program development, and this evidence informed ongoing change. Stakeholder input was high with keen interest in the development of the program from the various units and families on the ward.

### 2.4. Phase 4: Program Implementation

Phase four of the action research cycle involved clinical implementation of the program with the incorporation of various required changes in the process. Stakeholder consultation and collaboration had a large impact in this stage. Education sessions were provided in the clinical units to facilitate understanding by the staff of the processes involved and also to offer an opportunity for staff to express concerns or other feedback including suggestions for improvement. Three sessions were run in each unit. A Heart Beads resource folder was created containing the printed materials from education sessions, distribution forms, consents, and a journal for frontline ward staff to record candid comments and feedback from children, families, and other staff members relating to the program. Regular emails were sent to staff to ensure that they were informed about new developments and program feedback. Staff were also encouraged to use this mechanism to provide feedback and reflections to the project team.

The Heart Beads program was launched in July 2008. Participation in the program commences at the cardiac preadmission clinic and continues as the child is admitted to the ward. Families are offered the opportunity to be involved in Heart Beads and those wanting to participate complete a written consent. The child (or parent if the child is too young) is given a set of beads with the child's name spelt out and some of the beads for initial procedures planned. The beads are small porcelain shapes of varying colours and patterns that can be threaded onto a string. Families are also given an information flyer which outlines the purpose of the program and specifies what each of the distinct beads is for and how their child will access the beads during their stay in hospital. A distribution form or script is provided to the family so that they can continue collecting beads for the duration of the child's hospitalisation.

 The program evolved after consultation with the units involved and led to the development of individualised PICU and NICU distribution forms. As it is common for the child to progress through different units before arriving on the ward, a uniform process was developed. The distribution form was developed to allow the nurses to tick off procedures for which the child could collect a bead. The forms are given to families at the clinic so that once they return to hospital for admission, they can continue collecting beads when they are transferred to the PICU postsurgery or in the NICU if they are admitted there. Rather than beads being dispensed in every unit, it was decided to confine this part of the process to the ward. The form is carried through PICU with the child, and the parents are encouraged to prompt staff to sign off and date it once procedures/events have taken place. When the child is transferred to the cardiac ward, a new form is given to the parents listing more tests for which the child can receive beads. Parents go to the ward to receive the beads on the form even while the child is still in the PICU.

Initially, some resistance about the program was received from a small number of staff in the clinical areas concerned about whether it would add to their workload and what benefit it would be to the child and family. However, through education and witnessing the passionate reactions of children and families, they were ultimately able to see that the program added value rather than being an onerous task.

There was some dissatisfaction expressed by ward staff relating to the restriction on the number of people able to distribute the beads. The ward staff were reassured that this was not a trust issue, that it was related to the Risk Management Plan and that to ensure the continuation of organisational support the restriction needed to be adhered to. Staff were encouraged to be involved in the process of bead distribution by accompanying the child, young person, or parent when the script was redeemed through a designated person. Nurses were then able to maintain involvement in the program and had additional opportunities to communicate with children, young people, and parents in relation to their procedures and their experiences of them. The issues that arose during implementation were able to be taken into account and the program modified accordingly by utilising the knowledge gained through practice. Processing of bead distribution forms was further refined, new beads were added to recognise procedures as requested by other units and families.

### 2.5. Phase 5: Evaluation

The methods for program evaluation were determined after consultation between members of the project team (including the project officer, the cardiac NUM, and external research staff). Program evaluation, for the purpose of this report, involved staff and family interviews, process notes from the project officer, and anecdotal accounts from ward staff.

The Heart Beads program had been running for one year when formal evaluation, in the form of interviews, commenced in 2009. Purposive sampling was used for the interviews, and every attempt was made to include fathers, children, and young people where possible. Seven staff (including ward clerical and nursing staff) and eleven families consented to being interviewed after the commencement of the program. These interviews were conducted in a private room on the ward with a researcher and ranged from ten- to thirty-minute duration. Interviews were conversational in nature and were audiorecorded and transcribed. Interviewees were informed that the researchers (whom they had not previously met) were there to ask them about their experiences of the Heart Beads program and were provided with an information sheet explaining the evaluation. Interviewees were asked for their impressions of the Heart Beads program, what the program meant to them and their perceptions of the program overall. Interviews were coded and themed independently by two researchers, themes were discussed, and final themes agreed upon. Themes from family interviews have been discussed extensively in Redshaw et al. [30]. 

The project officer documented staff feedback, details of correspondence, project meeting notes, and other relevant information as process notes. These process notes, particularly relating to staff feedback, were used to continually refine the Heart Beads program. Continual review of the action research process, identifying issues and barriers and taking action to address these (e.g., addressing concerns about ongoing supply of the beads beyond the project) enabled the project team and staff on the ward to maintain momentum. Frontline nursing staff wrote up anecdotal accounts of the experiences of patients and their families involved in the project into a ward Heart Beads journal. The information from the process notes and anecdotal accounts, which was reviewed by researchers, has been brought together with the outcomes for staff and families. Qualitative data from the project thus includes interviews with staff, children, and families, analysed and themed following a grounded theory approach, and process notes and accounts from ward staff. It is not the purpose of this paper to discuss the findings of these interviews as these outcomes have been reported elsewhere. Briefly, outcomes included enthusiasm and excitement about the program, beads assisted with documentation of procedures and provided a record of experiences that impressed on staff and families the extent of what the child endured and the distress this involved. Evaluation of the Heart Beads program is ongoing and will continue for the duration of the program to ensure that the program meets its objectives and continues to enrich the hospital experience of children with cardiac conditions.

## 3. Discussion

The Heart Beads action research project involved a series of reflective cycles and continual evaluation [21], allowing the creation of a practical, effective program to be conducted with cardiac patients within the hospital [19, 22]. The continual reflection and evaluation involved in the project allowed relevant changes to be made and materials and processes refined, resulting in a more effective and sustainable program.

The action research approach as outlined by McNiff et al. [31] resulted in a partnership between clinicians who worked in the cardiac unit and researchers who offered research expertise, while also emphasising the involvement of other staff and families on the ward and from other units involved in cardiac procedures. This collaborative relationship is an essential component of action research enabling sustainable change, both to patient care and the work environment [23], as shown in the Heart Beads program and emphasised in other programs utilising this approach [14, 15, 17, 19, 20].

The action research approach was effective in bringing about the involvement of staff in the program and its continual enhancement and the development of their insights in relation to the care they provide. Increasing staff awareness of the experiences of the children in their care is imperative in paediatric nursing [32]. Providing a method such as the action research approach through which the program continues to be enhanced, to enable an increased understanding of the child's experience, can only benefit the care the hospitalised child and family receive, and ultimately their overall experience [33]. Evidence has emerged through staff team building exercises that the Heart Beads program is seen as an emblem of the common goals of the staff from the different units. The program has united the cardiac teams from preadmission clinic staff to the cardiac ward staff and allowed for clarification and strengthening of the values of the cardiac team. The program has enabled staff to engage more positively with the children and their families, which in turn facilitates enhanced therapeutic relationships. 

 Details of the Heart Beads program, particularly relevant outcomes, have been communicated throughout the hospital and at local, national, and International conferences. Scott [34] emphasises the importance of nursing research utilisation, highlighting that only a small proportion of research findings are actually translated into routine clinical practice. In contrast, the positive experience of Heart Beads families has attracted the interest of a cardiac support group, and the program is now being implemented nationwide with the vision that all Australian children hospitalised with cardiac conditions can benefit from Heart Beads.

## 4. Conclusion

The Heart Beads program began as a small project in the cardiac ward and continues to grow in popularity and significance. Throughout the development, implementation, and evaluation process, the Heart Beads program encountered numerous setbacks and challenges, many of which were overcome using the action research approach, as well as witnessing inspiring results and enthusiasm. Due to the overwhelmingly positive reactions from staff, children, and families alike, program continuation appears likely.

 The value of bead programs as a tangible tool to facilitate reflection and dialogue surrounding children's and families' hospital experiences is clearly acknowledged. The program has highlighted the importance of recognising the experiences of patients and families. Heart beads have allowed patients, families, and staff to appreciate the journey of children hospitalised with cardiac conditions.

Undertaking the project of developing, implementing, and evaluating the Heart Beads program through an action research approach has meant greater involvement of staff in the shaping and running of the program. Issues that have been raised in the process have been addressed and workable solutions found to ensure that the program remains effective and sustainable. Collaboration with researchers and with families provided opportunities for reflection and feedback from outside the ward environment, thereby enhancing program success.
